# Empirical Research on Male Preference in China: A Result of Gender Imbalance in the Seventh Population Census

**DOI:** 10.3390/ijerph19116482

**Published:** 2022-05-26

**Authors:** Yanzhe Zhang, Bowen Zou, Huai Zhang, Jian Zhang

**Affiliations:** 1 Northeast Asian Research Center of Jilin University, Changchun 130021, China; yanzhe_zhang@jlu.edu.cn; 2 Northeast Asian Studies College, Jilin University, Changchun 130021, China; bowen.zou2021@gmail.com (B.Z.); huai.zhang2022@gmail.com (H.Z.); 3 School of Northeast Asia, Jilin University, Changchun 130021, China; 4 School of International Economics and International Relations, Liaoning University, Shenyang 110136, China

**Keywords:** Seventh National Population Census, gender imbalance, male preference, China

## Abstract

The Seventh National Population Census, recently conducted in 2020, reported the most up-to-date information on the size, structure, and distribution of China’s population. The results showed that the gender imbalance in China is still severe compared with the international standard. With the aim of understanding what has contributed to China’s gender imbalance, this study examined a range of potential influencing factors and measured the extent to which they have affected China’s sex structure. We gathered data from 3100 citizens (100 surveys from each provincial-level administrative region in mainland China); the useful response rate was 87.5% (2713/3100). We relied on statistical analysis to investigate the phenomenon of male preference in China and used a logit regression to analyze the factors associated with this result. We inspected the factors associated with the perception according to gender, age, annual income, living location, educational level, nationality, family contribution, the ideology of being supported by sons, social status, ability to generate money, and carrying on the family name. The results showed that, among these factors, the relationship of family contribution, the ideology of being supported by sons, and carrying on the family name with male preference was significant. This study is among the first to explore the factors affecting male preference that could have resulted in China’s gender imbalance. The findings of this research are also important as references for the development of the population strategy and policy instruments used to manage the demographic problems in China.

## 1. Introduction

Since the founding of the People’s Republic of China, the Chinese government has conducted a national population census seven times as an important means of ascertaining the basic status of China’s populace [1]. Launched by the Chinese government, the Seventh National Population Census (taking place at 00:00 on 1 November 2020 as the reference time point) thoroughly investigated changes in the population characteristics and in housing conditions [2,3]. The respondents of the population census consisted of native persons within and Chinese citizens temporarily outside the territory of the People’s Republic of China, excluding people from overseas who were staying in the People’s Republic of China provisionally [4]. The population census mainly collected basic information on the populace and households, including name, personal ID number, gender, age, nationality, educational level, occupation, migration, marital status, fertility status, death, and housing [3]. It utilized electronic data collection and reported the data in real time through the autonomous submission of information by the respondents via a QR code for the first time. This promoted the use of big data by administrative agencies and improved the quality and efficiency of the work. The Seventh National Population Census randomly selected 32,000 households from 141 counties in 31 provinces (autonomous regions and municipalities directly under the central government) to carry out a subsequent investigation, and these results showed that the omission ratio of the population census was 0.05%, indicating a rigorous process and reliable results [2,4]. The results from the population census provided statistical data for use in China’s demographic development strategies and policies, as well as in the formulation of programs for a high standard of social and economic development.

**Figure 1:**
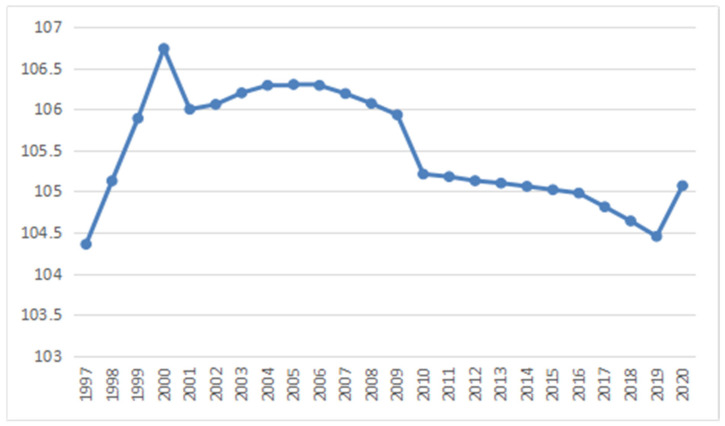
Gender ratio of China’s population, 1997–2020 (female = 100).

According to the data of the Seventh National Population Census, China is still confronted with the problem of gender imbalance in that there are more males than females [4]. The gender ratios of China’s whole population and newborn population experienced a period of gradual rise and then a continuous decline [4,5]. The communique of the Sixth National Population Census released by the National Bureau of Statistics pointed out that China had a population of 1332.81 million in total by the end of 2010, with males accounting for 51.27% and females accounting for 48.73% [4]. The gender ratio of the total population was 105.2 and the gender ratio of the newborn population was 118.06, which were both higher than the theoretical value (102–107) defined by the UN [2]. The results from the Seventh National Population Census indicate that China has a total population of 1411.78 million, and males and females comprise 51.24% and 48.76% of the total, respectively [3]. The gender ratio in 2020 dropped to 105.07, which was slightly lower than that in 2010 but was still higher than the world level in 2020 (see Figure 1) [6].

**Figure 2:**
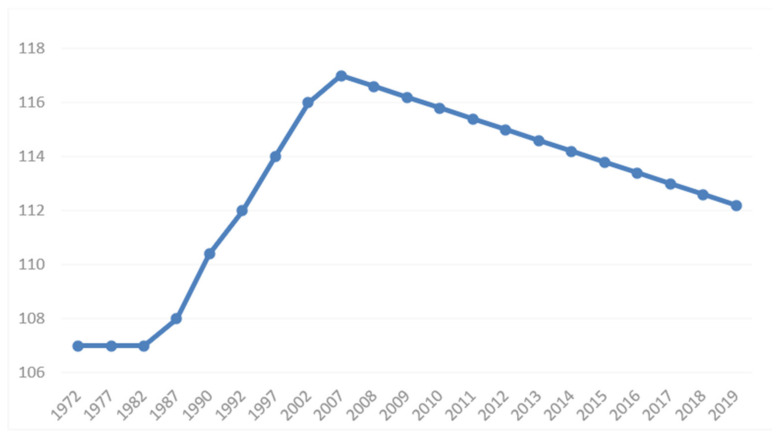
Gender ratio of China’s population at birth, 1972–2019 (female = 100) [9].

Moreover, data from the World Bank show that the gender ratio of the population at birth in China has been falling since 2007, incrementally dropping from 117 in 2007 to 112 in 2019 (see Figure 2) [7,8,9]. The communique of the Seventh National Population Census stated that the gender ratio of the newborn population was 111.3 in 2020, 6.8 lower than that in 2010 [4] but still far higher than the standard level set by the UN.

**Figure 3:**
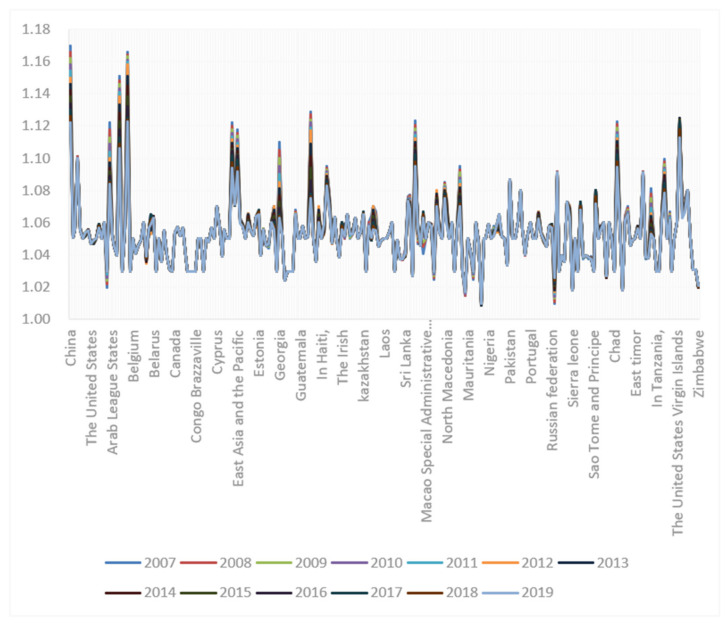
Gender ratio of population at birth by country, 2007–2019 [11].

In fact, China’s gender ratio at birth was relatively high among all countries across the world from 2007 to 2019, making China a country with typically more men than women. China’s Gender-Related Development Index (GDI) stood at 0.682 in 2020, ranking 107th out of 156 countries and regions, according to the *Global Gender Gap Report 2021* (see Figure 3) [3]. Human Development in 2020 also suggested that China’s Human Development Index (HDI) in 2019 was 0.761, ranking 85th out of the 189 countries and regions covered [10].

The reasons for this demographic problem are complex. Ding and Hesketh believe that China’s one-child policy has contributed greatly to the severe gender imbalance [12]. Ebenstein and Leung also emphasized that the limitation on births dictated by political pressure and the strong desire for male offspring have jointly led to parents making a gender selection [13]. The high gender ratio is ascribed to political factors; however, there are almost certainly social factors that have contributed to this demographic problem. Moreover, the ideology of privileging men over women plays an important part in China’s gender imbalance ratio at birth [14,15]. First, China’s patriarchal society, shaped by Confucian culture, puts males in a paramount position, and most families expect males for the continuity of the clan [16]. The core idea of ensuring the continuation of the family tree and the idea of “carrying on the family line with the male name” supports the traditional ideology that a male is always a better option than a female. Second, the idea of raising sons to support parents in their old age is a traditional concept of solving the economic problems linked to old age by raising children, especially males [17,18,19,20,21]. In China’s rural areas, insurance coverage for the elderly is still considerably limited, and the elderly population is mainly supported by their children. As a consequence, this outdated concept is still popular in rural communities. Third, Chinese history is based on the development of an agricultural civilization. Because of their advantages in strength, men have predominated in agricultural activities [22,23]. Although the social status of women has risen significantly due to economic development, they do not have complete equity in the workplace at present. At the same educational level and age, men are likely to earn more than women [24,25,26]. Furthermore, unlike men, who can devote themselves to their careers, working women are usually impacted by marriage, family, and childbearing, which may eventually affect their vocational development [27,28,29]. Worse still, enterprises also prefer male employees to female ones [30,31,32,33]. Therefore, Chinese society tends to prefer males in gender selection for practical reasons.

In summary, our aim was to contribute empirically and prescriptively to the contemporary research on the demographics of China. Few studies have been published from this perspective, as most previous studies have been limited by their narrow epistemological perspectives of family structure, the function of the social security sector, the process of implementing public health policies, etc., in exploring the burden on both society and the government in China. This highlights the absence of an investigation into the issues of male preference and factors causing the phenomenon of gender imbalance in China. The second main aim of this work was to explore why these issues have resulted in male preference and how they have become a key element that negatively affects the gender imbalance and rational development of China’s populace. The third and final contribution of this study lies in its insight into the demographics of China. Hopefully, it can guide Chinese policymakers to produce more effective policy instruments to deal with demographic issues.

In this study, both quantitative and qualitative approaches were used to assess the social factors that influence male preference and the gender imbalance in China. The quantitative analysis was based on feedback from surveys that collected data on gender, age, annual income, living location, educational level, nationality, family contribution, the ideology of being supported by sons, social status, the ability to generate money, and carrying on the family name. The qualitative analysis was based on data from interviews with political elites, including senior government officers, professors, and experts in the government. With regard to social issues, feedback from the surveys, depending on the willingness of the Chinese citizens who participated, and answers from the interviews with the political elites were used to understand the demographic characteristics of Chinese citizens. Both groups will influence the future population trends and demographic development of China.

## 2. Materials and Methods

### 2.1. Demographic Data

We obtained demographic data from the Seventh National Population Census in 2020. The National Population Census has been carried out approximately every 10 years (except for the 1970s), and was conducted in 1953, 1964, 1982, 1990, 2000, 2010, and 2020. It serves as an important means by which to ascertain the status quo of China’s demographic situation, including up-to-date information on the size, structure, and distribution of China’s population. After an analysis of the relevant data, the results of China’s Sixth National Population Census in 2010 indicated that China was faced with the realities of an aging population and gender imbalance. To this end, in 2011, the Chinese government proposed the two-child policy, which allowed couples who met certain conditions to have a second child. In November 2011, the two-child policy for families of only two children was implemented across China; in December 2013, the Chinese government put the two-child policy for families of one only child into practice; and in October 2015, China enacted the universal two-child policy, which allowed every couple to have two children. However, the results from the Seventh National Population Census in 2020 showed that the aging of the population and the gender imbalance have clearly not improved. In response, China has started to implement a three-child policy. On 31 May 2021, the Central Committee of the CPC held a conference and pointed out that “In order to further optimize the childbearing policy, a policy allowing every couple to have three children and supporting measures shall be implemented.” Adjustments to the policy will have a great influence on population trends and the gender imbalance.

### 2.2. Data Sampling

As the results from the Seventh National Population Census were released by the Chinese government at the end of May 2021, relevant research and analysis of the current demographic conditions exploring the factors that have resulted in a gender imbalance in China are absent. Thus, this research aimed to fill these gaps in the literature.

In June 2021, we initiated a survey named “An Assessment of Male Preference in China.” The aim of this investigation was to explore the factors that have resulted in the phenomenon of having more men than women in China (see Supplementary Materials). We collaborated with the National Bureau of Statistics, the National Health Commission of the People’s Republic of China, the Ministry of Civil Affairs of the People’s Republic of China, the National Development and Reform Commission, and the relevant governmental sectors to generate this questionnaire. The data collected in the survey included information related to gender, age, annual income, living location, educational level, nationality, family contribution, the ideology of being supported by sons, social status, the ability to generate money, and carrying on the family name (see Table 1).

The questionnaire was administered to 3100 individuals who had participated in the Seventh National Population Census. According to the geographical distribution of the population census (10 provincial administrative regions in Eastern China, 6 provincial administrative regions in Central China, 12 provincial administrative regions in Western China, and 3 provincial administrative regions in Northeast China), 100 surveys were assigned to each provincial administrative district, as 1000 of the 3100 participants were from Eastern China, 600 were from Central China, 1200 were from Western China, and 300 were from Northeast China. We received 2713 effective responses, resulting in an effective response rate of 87.5% (2713/3100). Data from the remaining 387 participants were absent due to meaningless responses and missing records. The authors examined two initial research questions (RQs):

RQ1. To what degree do Chinese citizens have a preference for males?

RQ2. What is/are the main factor(s) strongly associated with the gender imbalance in China?

Given the results of the interviews with the political elites, we also investigated a third research question:

RQ3. Are the political elites and policymakers aware that a gender imbalance will be harmful to social development in China?

### 2.3. Definition of the Variables

Table 2 shows that there were 2713 effective responses to the survey. The participants were enthusiastic and wanted to share their ideas about the results of the Seventh National Population Census and the phenomenon of gender imbalance in China. Among the most useful feedback was that 51.3% of respondents were male (1393) and 48.7% were women (*n* = 1320).

All of the participants involved in the surveys were adults and were over the legal marriageable age. In total, 21.4% of the effective participants (578 people) were aged 20–29 years, 20.7% (562 people) were aged 30–39 years, 20.9% (568 people) were aged 40–49 years, and 22.4% were aged 50–59 years (609 people). We received 396 effective responses (14.6%) from people aged over 60 years.

According to conventional recognition, families with lower annual incomes may have a greater need for males as future labor sources, which may be more prevalent in rural families [20]. Females may be more acceptable to urban families with higher incomes. The annual incomes of the participants in the survey were divided into six categories: CNY 0–99,999 (29.9%), CNY 100,000–199,999 (25.9%), CNY 200,000–299,999 (22.3%), CNY 300,000–399,999 (14.9%), CNY 400,000–499,999 (5.2%), and over CNY 500,000 (1.8%).

The gap between rich and poor in different parts of China is wide. The eastern seaboard is richer than the central region, which is richer than the west [12,14]. We believe that male preference is related to family wealth, which would lead to different geographical distributions, and we also explored this aspect. Thus, we assessed data from four regions: Eastern China (874 effective responses, 32.2%), Western China (998 effective responses, 36.8%), Central China (574 effective responses, 21.2%), and Northeast China (267 effective responses, 9.8%). Eastern China refers to Beijing, Tianjin, Hebei, Shanghai, Jiangsu, Zhejiang, Fujian, Shandong, Guangdong, and Hainan; Central China consists of Shanxi, Anhui, Jiangxi, Henan, Hubei, and Hunan; Western China is made up of Inner Mongolia, Guangxi, Chongqing, Sichuan, Guizhou, Yunnan, Tibet, Shaanxi, Gansu, Qinghai, Ningxia, and Xinjiang; and Northeast China contains Heilongjiang, Jilin, and Liaoning.

From the perspective of educational level, 579 people (21.3%) had reached only high school or lower. There were 1011 people (37.3%) with effective responses who had graduated from college. In total, 933 people (34.4%) who participated in the survey held a Bachelor’s degree, while 190 people (7.0%) had obtained a Master’s degree or above. In fact, male preference seems to be more tied to traditional beliefs, which we think might be mitigated by modern education. At the same time, modern education may improve the income and risk resistance of the educated group, thus further reducing the male preference of the educated group [30]. Therefore, we believed that this variable would be closely related to male preference.

China has many ethnic minorities. However, ethnic minorities may differ greatly from those of Han ethnicity in male preference due to their own traditions and common religious beliefs [11,15]. In general, religion may be more conducive to ethnic minorities’ male preferences. There were 1870 people (68.9%) of Han ethnicity who participated in the survey; the remaining 843 people (31.1%) were from ethnic minority communities.

Among the 2713 responses relating to a man’s contribution to the family, 259 people (9.5%) answered that it was none or a little, and 391 people (14.4%) considered that it was not much. The attitudes of 647 people (23.8%) were neutral. There were 1016 people (37.4%) who responded that a man’s contribution to the family is influential, and 400 respondents (14.7%) strongly believed that a man’s contribution is very important to family circumstances. In China, there is a traditional view that a married daughter is a member of her husband’s family and has separated from her family of origin. This stereotype often leads to a belief that males contribute more to the family and, in extreme cases, discrimination against daughters [21]. Modern ideas advocate more equality between men and women. We think this is a valid variable for measuring people’s male preference.

There is a belief in China that a son is a successor to his father’s career. The idea is very old. As early as the Ming Dynasty, there were laws stipulating that all the men in a family should do the same job [23]. More skilled traditional skills are also expected to be passed down to males. At the same time, modern families often hold the belief that successful males can effectively support, carry on, and carry forward their fathers’ careers [28]. We believe that this is closely related to male preference and that families with more traditional values regarding family ambition are more likely to prefer males. Of the responses related to providing support to the elderly, 8.8% (240 people) thought that sons provide little or no support to their elders, 20.5% (555 people) believed that sons do not make any effort to provide support to their elders, 680 people (25.1%) were neutral, 809 people (29.8%) partly agreed that a man can provide support to his elders, and 429 people (15.8%) agreed that a man provides great and positive support to his elders.

Moreover, respondents may choose to have sons because they think men have a higher social status in society or that it is more dignified to have a son [5,22]. Hence, 177 people (6.5%) believed that the social status of men is not higher than that of women, 346 (12.8%) people indicated that it was not too much higher, 640 (23.6%) gave a neutral response, and 910 (33.5%), from a total of 2713, considered that the social status of men is slightly higher than that of women in Chinese society. Additionally, 640 people (23.6%) believed that the social status of men is much higher than that of women in China.

Earning ability is an important criterion of success in modern Chinese society. More often than not, the financial burden of supporting aging parents and providing a good quality of life for the family they have created falls more heavily on men. A woman can choose a job earning CNY 4000–5000 per month, but if a man chooses such a job, he may be considered unambitious [14]. If the family of origin is poor, then males’ earning power expectations may be greater, leading to male preference. Therefore, this is an important variable. Therefore, in response to the question regarding ability to generate money, 140 people (5.2%) considered that the ability of men to generate money was little or none, 556 people (20.5%) thought that the ability of men to generate money did not have much importance, 822 people (30.3%) were neutral, 874 (32.2%) believed that the ability of men to generate money has some effect on male preference, and the remaining 321 people (11.8%) strongly believed that the ability of men to generate money influenced the preference for males in Chinese society.

The family name carries great significance in China. Family names have been passed down for thousands of years in a large family, indicating a common ancestry [15,27]. Unlike in the West, although a married woman does not have to change her surname to follow her husband, it is common for all children in a family to carry their father’s name. In families with a woman’s family name, the husband is often considered to live in a matrilocal residence and his family status is low. This situation often leads to male preference. Hence, of the survey responses related to carrying on the family name, the total number who believed that it was not important at all was 260 (9.6%), whereas 551 people (20.3%) considered that it was not very important. The number of respondents who gave a neutral answer was 701 (25.8%), whereas 789 (29.1%) stated that it is partly important and 412 (15.2%) maintained that it is very important.

### 2.4. Data Analyses

First, a descriptive analysis was used to determine the characteristics of the study population. The attitude of male preference was set as the dependent variable, and the responses for gender, age, LL, EL, ethnicity, FC, ISS, SS, AGM, and CFN were individually recognized as independent variables. The relationship between the dependent variable and the independent variables was analyzed using an ordered logit regression, as the dependent variables were categorized and ordered. When dealing with the relationships among discontinuous variables, logit regression can perform better than OLS regression. All of the statistical analyses were two-sided and carried out using SPSS (Version 17.0, SPSS Inc., Chicago, IL, USA) and STATA (MAC Version 14, Stata Co., College Station, TX, USA), and the statistical significance was set to *p* < 0.05.

## 3. Results

### 3.1. Sample Characteristics

Among the survey responses, regarding the degree of male preference perceived by the survey participants, 148 useful responses (5.5%) rated it as none or little, 18.5% (501 people) answered “not much”, 614 people (22.6%) held a neutral attitude, 1020 people (37.6%) perceived some male preferences, and 15.8% of the 2713 useful responses believed that it was very high.

### 3.2. Quantitative Analysis

Table 3 provides the results of the ordered logistic regression analysis, including coefficients, standard deviations, *p*-values, and confidence intervals. First, we tested the fit of the model, and the results showed that the *p*-value (Sig.) was 0.041, indicating the overall significance of the model. Second, a test of parallel lines was conducted, and the *p*-value (Sig.) was 0.152, which is greater than 0.05, thereby satisfying the test of parallel lines. This indicates that the estimated parametric value of the ordered logistic regression model was reliable and reflected the influence of the independent variables in the model on the dependent variable.

We firstly find that no or little FC was significant at the significance level of 5%, with a coefficient of −0.302, that is, FC is positively correlated with male preference. Secondly, “no”, “little”, or “not much” ISS results were significant at the significance level of 1% and 5%, respectively, with coefficients of −0.399 and −0.281. This shows that the idea of sons supporting the family is positively correlated with male preference. Finally, “no”, “little”, or “not much” CFN results were both negatively correlated with male preference at the 1% confidence level, with coefficients of −0.416 and −0.423. This also indicates that the concept of carrying on family name has a positive influence on male preference, which is the same as our expectation. To sum up, as shown in Table 2, the significant relationships between the dependent variable of male preference and the independent variables of FC, ISS, and CFN were significant. In fact, the respondents of this survey affirmed that men can contribute more to their families and that they preferred males in order to continue their family line. Moreover, some respondents believed that a married daughter belongs to the man’s family, and that only sons can provide them with support in their old age. This is another reason why the respondents prefer male children. In addition, carrying on the family name was another significant factor that influenced the respondents’ male preference. However, the estimated coefficients of gender, age, AI, LL, EL, ethnicity, SS, and AGM had no statistical significance in this model (*p* > 0.05).

### 3.3. Qualitative Analysis

We conducted interviews with five elite officials. Two of them were professors of demographic studies at Hong Kong University and Renmin University, and another was a professor of Public Administration and Public Policy at Tsinghua University. In addition, two senior officers at the National Bureau of Statistics and the National Health Commission of the People’s Republic of China were interviewed. One of the authors conducted personal interviews with these officials at their workplaces. The questions related to the results of Seventh National Population Census and the Chinese demographic policy. The duration of the interviews was approximately 1–2 h. The author was permitted to take notes in English.

As expected, the interview reports reflected what had been indicated in the qualitative analysis, by and large. The interviewees all held the idea that the gender imbalance, as one of the major challenges of China’s further economic and social development, is a looming problem, and that all of Chinese society would be doomed to suffer great loss and face intractable social instability if officials simply let things drift. Male preference is derived from ancient agricultural society, and two reasons may explain the concept. First, males, by virtue of their physical advantages, were more suitable for manual labor and agricultural production, which accounted for the bulk of household income. Military activities to maintain local security also required the large-scale participation of men. Second, as taught by Confucian culture, China’s patriarchal society placed a premium on carrying on the family line; in most cases, only male children were considered to be the heirs of the clan. These can be summed up as economic and political factors as well as cultural factors.

The full introduction of one-child policy in the 1980s forced many Chinese families into gender selection. Unlike self-determination within a family, this was probably the first time in Chinese history when limits on births were imposed via a national policy. Under such circumstances, male preference directly led to China’s loss of “half a generation of women”. Since the reform and opening up in 1978, China has witnessed its economy soaring over the past decades, and agriculture is no longer China’s pillar industry. However, Chinese families’ preference for male offspring has not been attenuated by the abolishment of the one-child policy and economic progress, indicating that cultural influences rather than economic or political factors shaped the general tendency for male preference across Chinese society. This was basically consistent with the results of the quantitative analysis: compared with gender, age, and ethnicity, which focus on basic individual information, and AI, AGM, and LL, which consider economic factors, the dependent variables concerning cultural traditions, such as FC, ISS, and CFN, made a very large contribution to the consequence of widespread gender selection.

## 4. Discussion

The results of the Seventh National Population Census showed what is at the center of the current situation regarding China’s demographics. It has been recognized as an official parameter that can provide references for the formulation of a long-term policy strategy and instruments for demographic and social development, as well as information to aid in the promotion of high-quality economic growth in China [1,4]. An increasing number of researchers have begun to investigate and evaluate the outcomes and results of the Seventh National Population Census in China [2,5], but few studies have explored what factors led to the current demographic circumstances in China, especially the very significant problem of gender imbalance. The present study is one of the first attempts to conduct an analysis of the current situation in China’s population and to further examine the social issues influencing the demographic characteristic of gender imbalance in China. The findings of this research provide new insights into demographic studies in China. The results of this study are important for use in the development of demographic strategies and for guiding elites in initiating a population policy instrument in China.

Chinese history and society have developed from an agricultural civilization that produced the ideology of privileging men over women. First, the common value of male preference has significantly affected Chinese society with regard to gender selection and has resulted in the phenomenon of gender imbalance. Policy interventions could place an emphasis on supporting the rights and interests of women, especially for females in rural areas. It has also been suggested that the government should look after women, including providing more opportunities for education and jobs. For example, Hubei Province has adopted the policy of giving bonus points in college entrance examinations to females from rural, single-child families. According to the policy, since April 2009, all eligible females have been able to earn 10 extra points towards the total score [34]. This policy, to a certain extent, promotes a change in people’s concept of marriage and childbirth, and effectively addresses the high gender ratio at birth. Furthermore, it maintains the interests of females from rural, single-child families in receiving an education and stimulates family investment in the education of females.

Second, consistent with previous studies, this study proposes that the ideology of male preference has had a significant impact on the demographic characteristic of gender imbalance. This may be because Chinese people have traditionally relied on men to support their families [35,36]. It is recommended that the Chinese government facilitate a set of policy instruments, including legislation, policy guidance, and publicity, to respect and protect women’s rights and interests in China. Under such circumstances, enterprises that provide more job opportunities to women could be offered a tax reduction by both the local and central governments.

Moreover, the traditional ideology of carrying on the family name is also contributing to the phenomenon of gender imbalance in China. This ideology is universal, but it has a unique interpretation in Chinese philosophy that there are three forms of filial impiety, of which having no offspring is the greatest [37,38,39]. The traditional concept of procreation is deeply rooted in the minds of most Chinese people, and carrying on the family name is integral to the happiness of families [40,41]. Moreover, males are needed to inherit the family fortune. Unlike some other countries, a Chinese woman does not have to drop her own surname and take that of her husband, but China’s traditional values believed that the females were not “rightful” successors of their clans under most circumstances, and children were not very likely to adopt their mother’s surname.

China implemented a “one-child policy” in 1979, but many families were willing to have more children at any cost in order to give birth to a boy, including having multiple births until a baby boy was born and gender selection by induced abortion [42]. Previously, gender-selective induced abortions were quite common in China. In 2003, the Chinese government issued the Regulations on the Prohibition of Sex Authentication for Non-medical Purposes and Attempts to Terminate Pregnancies for Gender Selection Purposes, which stated that “No institution or individual shall conduct fetal gender authentication and termination of pregnancy without the approval of the health administrative department and the family planning administrative department; fetal gender selection and pregnancy termination for non-medical purposes shall be strictly forbidden” [43,44,45]. However, there are still many cases of illegal induced abortions. According to survey data, on 1 July 2017, the gender ratio at birth of all samples was 112.13, the abortion ratio was 16.16%, and the gender-selective abortion ratio was 11.02% [46]. A study by La Trobe University in 2019 pointed out that some Chinese women specifically went to Australia for fetal gender authentication, and they would terminate the pregnancy as soon as they found they were carrying a female [4].

Factors relating to basic personal information such as gender, age, and ethnicity, according to the quantitative analysis, all had an insignificant effect on China’s male preference, indicating that the ideology of privileging men over women is considerably common in daily life. What is contrary to intuition, however, is that economic and political factors do not have a significant relationship with China’s gender imbalance. In general, respondents from different classes divided by economic conditions and social status all indicated a tendency towards male preference. This indicates a problem: women’s economic and political status has gradually increased with social progress, but they are still in subordinate positions when it comes to development and the continuity of family clans. This may also offer suggestions to China’s policy-making authorities. Before the founding of the People’s Republic of China in 1949, Chinese society was, on the whole, a huge agricultural community. After that, the development of grassroots political institutions incrementally undermined the patriarchal clan idea in China, especially in rural areas. However, it is not easy to transform the inveterate concepts within mere decades by political pressure and economic incentives. Culture evolves at a snail’s pace, and its persistence means that it will take a long time before such policies can really work [47]. It will be a protracted war to ameliorate the gender structure, but potential risks are hanging overhead. It is not wise to rely merely on policy instruments, especially economic ones. Propaganda and legal protection should also play key roles [48], so that China can begin the process of improving the gender structure.

The long-term gender imbalance in China has created many problems, the most direct of which are the potential social problems. The first of these is mental and physical health. The embarrassment suffered by single males is contrary to China’s marital tradition and childbearing culture. Faced with discrimination at work and in life, these men suffer from tremendous mental stress. Hu found that staying married reduced the likelihood of depression compared with single men [49]. Moreover, Ma collected data from 380 villages from 18 provinces in China and researched the relationship between men affected by “marriage crowding-out” and behaviors that threatened themselves and their social safety (here, marriage crowding-out refers to the imbalance between the numbers of males and females of marriageable age) [47]. Studies have shown that men affected by “marriage crowding-out” are more susceptible to certain dangerous behaviors, especially ones that threaten their safety, and their scores for suicidal thoughts were much higher than those of other men, particularly those of optimal childbearing age (28–49 years) [50,51,52]. Generally speaking, marriage crowding-out is not confined within a certain area, thanks to population migration and changes in the population’s age structure, but the situation can be more serious when the whole country is faced with a structural gender imbalance. As a result, there will always be a large group of bachelors. For these men, deprived of emotional affiliation and sexual fulfilment, the lack of a marriage and family to fulfill social functions, such as education, medical care, and carrying on the family line, can trigger a crisis of sexual repression and psychological distortion. Furthermore, anxiety about being excluded from marriage and passing away without offspring means that single men in poor and rural areas are vulnerable to marriage by deception. The huge market created by single males in less developed areas has also spawned crimes such as payment for marriage, as well as the abduction and trafficking of women and children. Furthermore, gender imbalance also tends to increase the risk of infection with AIDS and other sexually transmitted diseases [53].

Moreover, support of the elderly by a family member is one of the most significant traditions in China. Unless the gender imbalance can be kept within a reasonable range, more men will be deprived of an opportunity to marry, and they will have no choice but to rely on the government for care in their old age. Jin tested the negative effect on the rural retirement safeguard created by marriage crowding-out by surveying pension data in rural areas from the perspectives of gender, marital status, and life satisfaction [54,55,56]. The research showed that, for China’s elderly population (those over 60 years old), financial support payments from other family members are the most important source of income, accounting for 40.72% of the total compared with 47.7% in rural areas. This reflects the fact that relying on support from offspring is still a major pension source in Chinese families. Allowing for the failure to procreate, government relief is the main source of support for single males in their old age. This is a huge impediment to developing the national economy and society, and to boosting high-quality development in China.

## 5. Conclusions

The aim of this study was to explore the factors that have resulted in the phenomenon of male preference and the related demographic problems, such as gender imbalance, in China. Hence, three research questions were addressed in this study: (1) “To what degree do Chinese citizens prefer males?”; (2) “What is/are the main factors strongly associated with gender imbalance in China?”, and (3) “Are the political elites and policymakers aware that gender imbalance will be harmful to social development in China?” To answer the first question, we used descriptive statistics to determine the number of people who displayed male preference in China. The number of views opposing this was 649 out of 2713, as shown in the surveys; thus, 1450 people had the attitude of male preference in China, and 614 people’s attitudes were neutral.

To address the second question, we designed a survey entitled “An Assessment of Male Preference in China”. We used ordered-logistic regression to analyze the data, and found that the factors of family contribution, the ideology of being supported by sons, and carrying on the family name had a strong relationship with the phenomenon of male preference in China.

To answer the third question, we interviewed five elites, including professionals and senior officials. Their feedback was positive and constructive regarding the current demographic situation in China. They suggested Chinese families’ male preference and prejudice against females were blunt facts at the current stage, and the Chinese government should take the rights and interests of women fully into account in the process of policy making to weaken the tendency towards male preference and promote gender balance in China.

This study has several limitations. First, it was based on a quantitative research method. The data were generated from surveys of 31 out of 34 provincial-level administrative regions in China. We did not distribute the questionnaires to the respondents according to ethical considerations but according to geographical factors. This may mean that the results are not accurate or fully reflective. Further research is required to consider these issues, and to quantify more factors in order to produce more precise results. Second, this research was a study on the results of the Seventh National Population Census. These results reflect the continuous changes in previous demographic conditions. The results of previous population censuses were not emphasized in this study. This issue needs to be acknowledged in further studies. Third, some differences exist in the cognitive abilities of the different age groups. Fourth and lastly, this study contained interviews with political elites. These political elites were from both the central government and the top think-tanks in China, but did not include people who work at the local government level. Further studies are needed to obtain more information from political elites in different fields.

In conclusion, this research provided two main findings. First, we explored the proportion of those who have the attitude of male preference in China. Second, we observed that the factors of family contribution, the ideology of being supported by sons, and carrying on the family name have a strong influence on male preference, resulting in the gender imbalance in China. This may be the first comprehensive study of male preference, gender imbalance, and their significant associated factors in China. Our research has crucial implications for improving population development strategies and policies, promoting long-term balanced population development, formulating programs for the development of the national economy and Chinese society, and boosting high-quality economic development.

## Figures and Tables

**Table 1:** The list of variables in the questionnaire.

Variable	Code Name	Practical Significance
Gender	Gender	Gender of respondents.
Age	Age	Age of respondents.
Annual Income	AI	The annual income of the interviewee may be related to many factors, such as education level and open-minded thinking, and thus affect their male preference.
Living Location	LL	The traditional values of China’s rural areas are stronger than those of urban areas, and rural residents may be more inclined to have a boy as a family worker.
Educational Level	EL	Higher levels of education may eliminate male preference or discrimination against daughters among the educated.
Ethnicity	Ethnicity	Traditional beliefs and religious beliefs are more common among China’s ethnic minorities than among Han Chinese, which may further influence the respondents’ male preference.
Family contribution	FC	In the traditional Chinese concept, a married daughter is considered to be a member of another family. This often affects male preferences.
Ideology of being supported by sons	ISS	Many families have the idea that the adult son will be the main support of his parents. At the same time, families with a trade or business are more likely to want their sons to carry on the business. We propose this variable to measure the traditional Chinese expectation of sons supporting the family.
Social status	SS	Respondents’ judgments of the social status of men and women.
Ability to generate money	AGM	In Chinese society, men are often seen as the breadwinners of the family and have greater family responsibilities. A family’s desire for money may make them more likely to choose to have a son.
Carrying on the Family Name	CFN	Carrying on the family name is an ancient tradition in China. The family name can only be inherited by a son. Families without sons to pass on the name may be thought to be losing their family name. This undoubtedly affects male preference in the family.

**Table 2:** Definitions and descriptions of the variables included in the survey (*n* = 2713): gender, age, annual income (AI), living location (LL), educational level (EL), ethnicity, family contribution (FC), ideology of being supported by sons (ISS), social status (SS), ability to generate money (AGM), and carrying on the family name (CFN).

Variable	Measurement	Min.	Max.	Mean	Percentage *n*
Gender	1 = Male	1	2	1.49	51.3% (1393)
	2 = Female				48.7% (1320)
Age	1 = 20–29	1	5	2.88	21.4% (578)
	2 = 30–39				20.7% (562)
	3 = 40–49				20.9% (568)
	4 = 50–59				22.4% (609)
	5 = 60+				14.6% (396)
AI	1 = CNY 0–99,999	1	6	2.45	29.9% (810)
	2 = CNY 100,000–199,999				25.9% (704)
	3 = CNY 200,000–299,999				22.3% (604)
	4 = CNY 300,000–399,999				14.9% (403)
	5 = CNY 400,000–499,999				5.2% (142)
	6 = CNY 500,000+				1.8% (50)
LL	1 = East	1	4	2.09	32.2% (874)
	2 = West				36.8% (998)
	3 = Central				21.2% (574)
	4 = Northeast				9.8% (267)
EL	1 = High school-	1	4	2.27	21.3% (579)
	2 = College				37.3% (1011)
	3 = Bachelor’s degree				34.4% (933)
	4 = Master’s degree+				7.0% (190)
Ethnicity	1 = Han ethnicity	1	2	1.31	68.9% (1870)
	2 = Ethnic minorities				31.1% (843)
FC	1 = None or little	1	5	3.33	9.5% (259)
	2 = Not much				14.4% (391)
	3 = Neutral				23.8% (647)
	4 = Partly				37.4% (1016)
	5 = Very much				14.7% (400)
ISS	1 = None or little	1	5	3.23	8.8% (240)
	2 = Not much				20.5% (555)
	3 = Neutral				25.1% (680)
	4 = Partly				29.8% (809)
	5 = Very much				15.8% (429)
SS	1 = None or little	1	5	3.55	6.5% (177)
	2 = Not much				12.8% (346)
	3 = Neutral				23.6% (640)
	4 = Partly				33.5% (910)
	5 = Very much				23.6% (640)
AGM	1 = None or little	1	5	3.25	5.2% (140)
	2 = Not much				20.5% (556
	3 = Neutral				30.3% (822
	4 = Partly				32.2% (874
	5 = Very much				11.8% (321)
CFN	1 = None or little	1	5	3.20	9.6% (260)
	2 = Not much				20.3% (551)
	3 = Neutral				25.8% (701)
	4 = Partly				29.1% (789)
	5 = Very much				15.2% (412)
Male preference	1 = None or little	1	5	3.40	5.5% (148)
	2 = Not much				18.5% (501)
	3 = Neutral				22.6% (614)
	4 = Partly				37.6% (1020)
	5 = Very much				15.8% (430)

**Table 3:** Coefficients of the ordinal logit regression (*n* = 2713).

Variables	None or Little	Not Much	Neutral	Partly	Very Much	Coefficient (S.E.)	*p*-Value	95% CI
**Gender**								
Male	75	258	308	538	214	0.004 (0.070)	0.959	−0.134–0.142
Female	73	243	306	482	216			
**Age**								
20–29	33	102	156	225	62	−0.108 (0.121)	0.376	−0.346–0.131
30–39	31	106	118	214	93	0.084 (0.121)	0.490	−0.154–0.321
40–49	28	100	128	203	109	0.151 (0.121)	0.212	−0.086–0.388
50–59	34	121	119	227	108	0.077 (0.119)	0.517	−0.157–0.312
60+	22	72	93	151	58			
**AI**								
CNY 0–99,999	43	135	173	335	124	0.222 (0.268)	0.408	−0.304–0.748
CNY 100,000–199,999	34	162	164	220	124	0.022 (0.270)	0.935	−0.508–0.552
CNY 200,000–299,999	38	103	119	245	99	0.195 (0.270)	0.470	−0.334–0.723
CNY 300,000–399,999	25	63	113	145	57	0.029 (0.274)	0.916	−0.507–0.565
CNY 400,000–499,999	4	31	32	55	20	0.140 (0.299)	0.639	−0.446–0.726
CNY 500,000+	4	7	13	20	6			
**LL**								
East	47	156	204	323	144	0.103 (0.130)	0.429	−0.152–0.357
West	54	190	227	367	160	0.085 (0.126)	0.502	−0.162–0.332
Neutral	31	104	118	235	86	0.153 (0.135)	0.259	−0.112–0.418
Northeast	16	51	65	95	40			
**EL**								
High school-	33	111	124	216	95	0.112 (0.154)	0.467	−0.190–0.413
College	55	189	231	375	161	0.100 (0.145)	0.492	−0.185–0.385
Bachelor’s degree	49	161	218	352	153	0.171 (0.145)	0.238	−0.113–0.456
Master’s degree+	11	40	41	77	21			
**Ethnicity**								
Han	103	337	429	709	292	−0.005 (0.076)	0.947	−0.154–0.144
Ethnic minorities	45	164	185	311	138			
**FC**								
None or little	18	56	56	93	36	−0.302 ** (0.145)	0.037	−0.586-−0.018
Not much	22	71	82	142	74	−0.053 (0.130)	0.682	−0.308–0.201
Neutral	38	118	135	261	95	−0.162 (0.116)	0.163	−0.388–0.065
Partly	54	192	236	388	146	−0.191 (0.108)	0.076	−0.402–0.020
Very much	16	64	105	136	79			
**ISS**								
None or little	17	57	45	86	35	−0.399 *** (0.147)	0.007	−0.687–0.111
Not much	35	121	110	207	82	−0.281 ** (0.118)	0.017	−0.511–0.050
Neutral	31	131	159	253	106	−0.192 (0.112)	0.087	−0.412–0.028
Partly	49	129	201	296	134	−0.133 (0.109)	0.222	−0.347–0.080
Very much	16	63	99	178	73			
**SS**								
None or little	11	33	36	71	26	−0.011 (0.155)	0.943	−0.315–0.293
Not much	19	69	75	130	53	−0.051 (0.122)	0.675	−0.290–0.188
Neutral	35	116	143	243	103	0.020 (0.102)	0.844	−0.179–0.219
Partly	49	164	219	331	147	−0.016 (0.094)	0.863	−0.201–0.168
Very much	34	119	141	245	101			
**AGM**								
None or little	7	25	35	48	25	0.003 (0.186)	0.986	−0.361–0.367
Not much	31	105	120	214	86	0.008 (0.127)	0.947	−0.241–0.258
Neutral	44	152	179	314	133	0.004 (0.120)	0.971	−0.230–0.239
Partly	48	161	204	327	134	−0.038 (0.119)	0.747	−0.271–0.194
Very much	18	58	76	117	52			
**CFN**								
None or little	20	52	64	87	37	−0.416 *** (0.144)	0.004	−0.698–0.133
Not much	32	111	135	220	53	−0.423 *** (0.119)	0.000	−0.656–0.190
Neutral	29	115	159	283	115	−0.081 (0.113)	0.476	−0.302–0.141
Partly	49	147	172	286	135	−0.203 (0.111)	0.066	−0.420–0.013
Very much	18	76	84	144	90			
**Observations**	*n* = 2713							
**Df**	37							

Note: *** *p* < 0.01 and ** *p* < 0.05.

## Data Availability

Not applicable.

## Supplementary Materials

The following supporting information can be downloaded at: https://www.mdpi.com/article/10.3390/ijerph19116482/s1.
