# Supplementary material for: Empirical Research on Male Preference in China: A Result of Gender Imbalance in the Seventh Population Census
Source: Int J Environ Res Public Health. 2022 May 26;19(11):6482. doi: 10.3390/ijerph19116482 (PMC9180325; doi:10.3390/ijerph19116482)
Supplement: Supplementary file 1 [file ijerph-19-06482-s001.zip › ijerph-1726953-supplementary.pdf]

An Assessment of Male-preference in China

Hello! Thank you very much for participating in the survey about the 7th Demographic census. In May 2020, the 7th Demographic Census just released data showing that the phenomenon of imbalance gender is still severe in China.

This questionnaire is designed to understand your assessment in choosing genders. The questionnaire is anonymous. The results are only for scientific research and will not have any impact on you. Please feel rest to assured that the true answer! Choose the option that best suits your opinion under each question. Thank you for your support and cooperation!

Part One: Basic Information

1. Gender
   A: Male
   B: Female
2. Age
3. Annual Income
4. Living Location
5. Education Level
6. Nationality
7. Do you know the 7th Demographic Census in China? (If "Yes", Please go to the rest of this questionnaire. If "No", your questionnaire is complete).
   A: Yes
   B: No

Part Two: Questionnaire

8. To what degree you thought the male's contribution is important?
   A: None or few (0–20)
   B: Not too much (21–40)
   C: Neutral (41–60)
   D: Partially (61–80)
   E: Very much (81–100)

9. To what degree you thought the traditional ideology of supported by sons is right?
   A: None or few (0–20)
   B: Not too much (21–40)
   C: Neutral (41–60)
   D: Partially (61–80)
   E: Very much (81–100)

10. To what degree you thought the differences in social statue between male and female exist in China?
    A: None or few (0–20)
    B: Not too much (21–40)
    C: Neutral (41–60)
    D: Partially (61–80)
    E: Very much (81–100)

11. To what degree you thought the males' ability of generating money are greater than female in China?

A: None or few (0–20)
B: Not too much (21–40)
C: Neutral (41–60)
D: Partially (61–80)
E: Very much (81–100)

12. To what degree you thought the ideology of carrying family name is important in China?
    A: None or few (0–20)
    B: Not too much (21–40)
    C: Neutral (41–60)
    D: Partially (61–80)
    E: Very much (81–100)

13. To what degree you prefer boy as the first baby in your family?
    A: None or few (0–20)
    B: Not too much (21–40)
    C: Neutral (41–60)
    D: Partially (61–80)
    E: Very much (81–100)

14. To what degree you thought you are male-preference?
    A: None or few (0–20)
    B: Not too much (21–40)
    C: Neutral (41–60)
    D: Partially (61–80)
    E: Very much (81–100)

15. Any comments to further concerns of the gender imbalance in China's society?
